# Supplementary figures and images for: Epigenetic and transcriptomic reprogramming in monocytes of severe COVID-19 patients reflects alterations in myeloid differentiation and the influence of inflammatory cytokines
Source: Genome Med. 2022 Nov 29;14:134. doi: 10.1186/s13073-022-01137-4 (PMC9706884; doi:10.1186/s13073-022-01137-4)

Figure S1

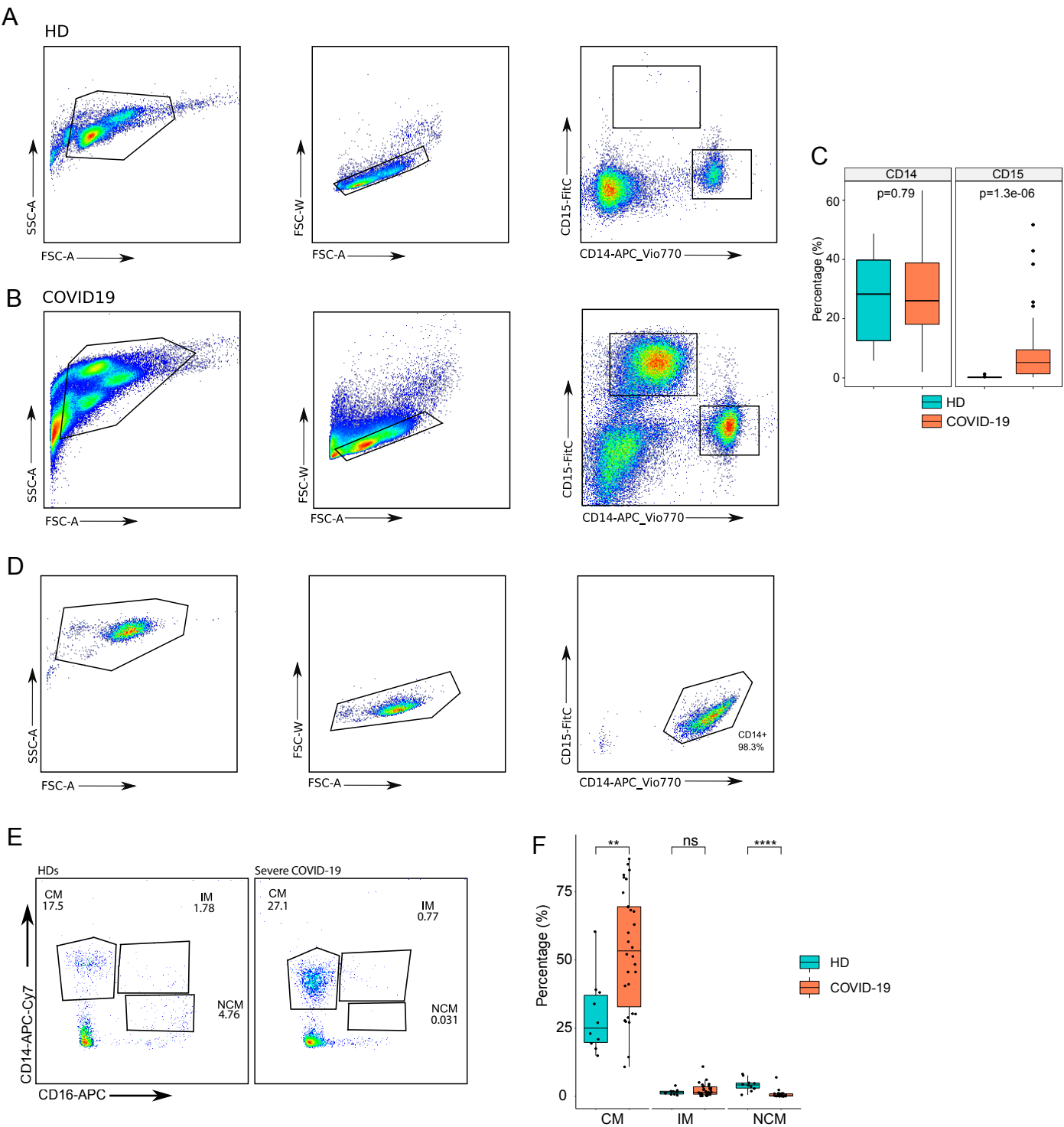

Supplement: Supplementary file 4 — Additional file 4: Figure S1. Flow cytometry profiles of a representative sample for each group; HD (A) and COVID-19 (B) indicating the sorting strategy and gates used in the study. C Boxplot representing the mean percentage of CD14+ and CD15+ cells in HD and patients. D Flow cytometry profile of the CD14+CD15- purified monocytes using the same gates used in the study. E HD and COVID-19 indicating the gates used for monocyte subtype analysis (classical monocytes, CM; intermediate monocytes, IM; and non-classical monocytes, NCM) (F) Boxplot representing the mean percentage of CM, IM and NCM in HD and COVID-19 patients. [file 13073_2022_1137_MOESM4_ESM.pdf]

Figure S2

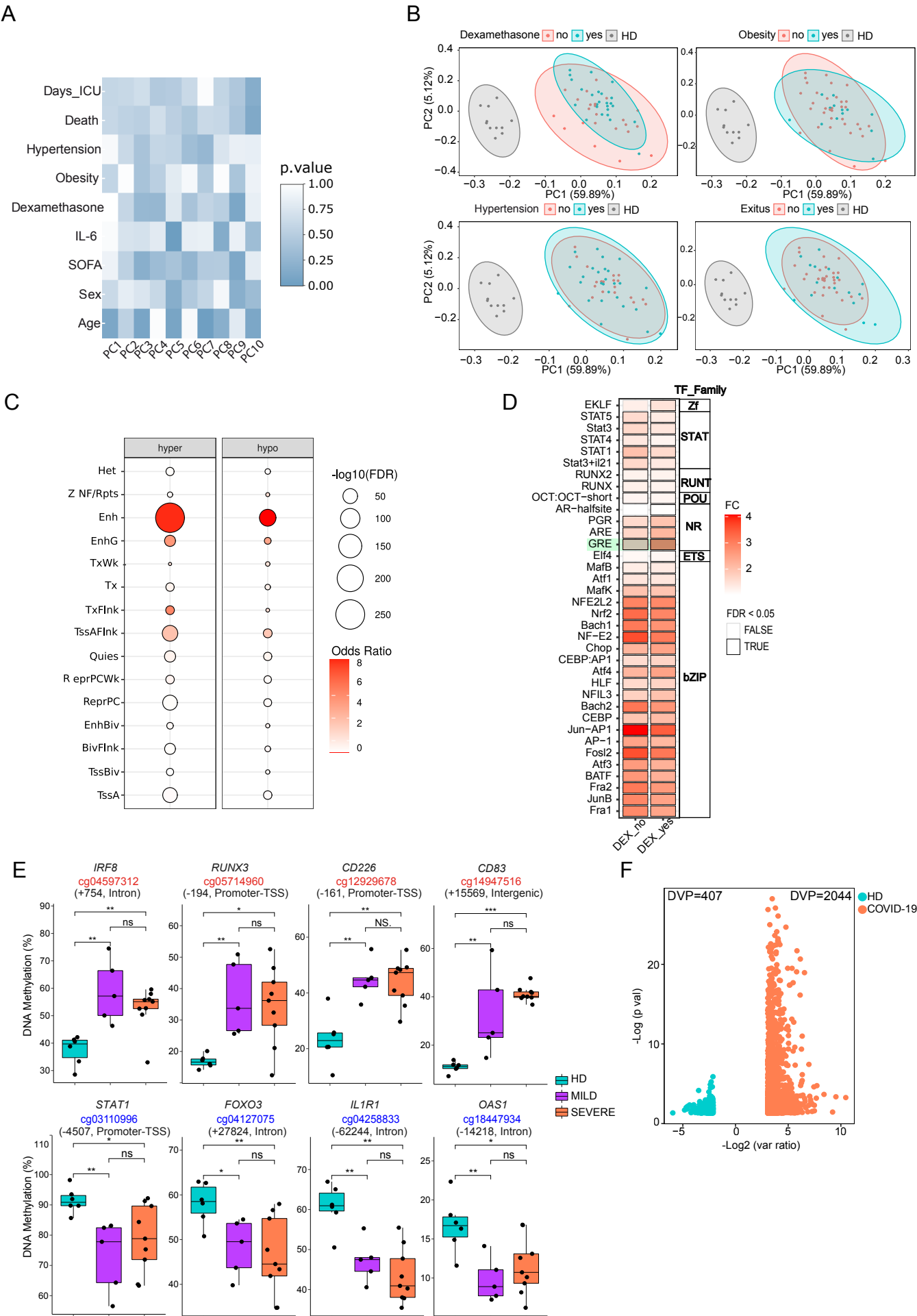

Supplement: Supplementary file 6 — Additional file 6: Figure S2. DNA methylation analysis in blood monocytes of severe COVID-19 patients. A Venn diagram of the overlap of hyper- and hypomethylated DMPs identified with a subcluster of samples including (CM/IM_YES) or not (CM/IM_NO) the CM and IM percentage as a covariable in the comparison between HD and severe COVID-19 patients (B) Heatmap representation of beta values of first 10 Principal component (PC) analysis that correlations PCs with different clinical variables. Numerical variables were correlated to PCs using Pearson correlation, whereas categorical variables were entered in a linear model together with the PCs. C Principal component analysis (PCA) of the DMPs. The HDs are illustrated in grey, and the severe COVID-19 patients are illustrated as blue and red in function of the clinical parameter or treatment with dexamethasone. D Enrichment analysis of different chromatin states for CpGs sites corresponding the Hyper- and Hypomethylated clusters. The FDR is represented with the size of the bubble, as shown. The relative enrichment is represented as Odds Ratio. TssA, Active TSS; TssBiv, Bivalent/Poised TSS; BivFlnk, Flanking Bivalent TSS/Enh; EnhBiv, Bivalent Enhancer; ReprPC, Repressed PolyComb; ReprPCWk, Weak Repressed PolyComb; Quies, Quiescent/Low; TssAFlnk, Flanking Active TSS; TxFlnk, Transcr. at gene 5' and 3'; Tx, Strong transcription; TxWk, Weak transcription; EnhG, Genic enhancers; Enh, Enhancers; ZNF/Rpts, ZNF genes & repeats; Het, Heterochromatin. E TF binding motif analysis of hypomethylated DMPs comparing patients no treated with dexamethasone vs. HDs (DEX_no) and patients treated with dexamethasone vs. HDs (DEX_yes). The panel shows the fold change (FC), TF family. Black outlined boxes indicate TF binding motifs with FDR values < 0.05. F Box plot of individual DNA methylation values of CpG from the hypermethylated and hypomethylated clusters with the name of the closest gene and the position in respect to the transcription start [file 13073_2022_1137_MOESM6_ESM.pdf]

Figure S3

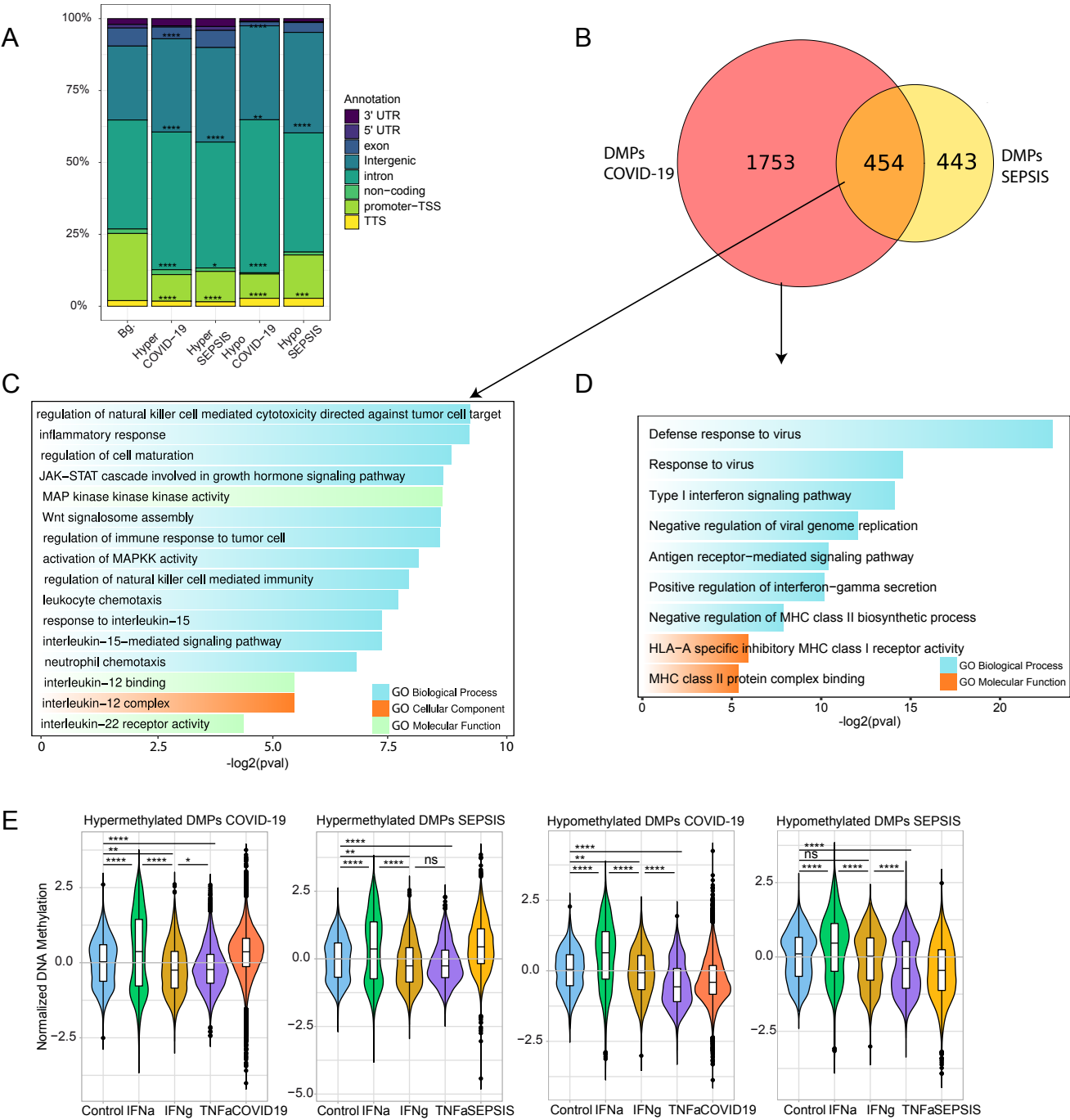

Supplement: Supplementary file 8 — Additional file 8: Figure S3. DNA methylation comparative analysis between blood monocytes of severe COVID-19 patients and bacterial sepsis patients (A) Proportions of the genomic locations (in relation to genes) of hyper- and hypomethylated DMPs in COVID-19 and sepsis; Bg., background, EPIC probes. B Venn diagram of the overlap of COVID-19 DMPs identified by the comparison between HD and severe COVID-19 patients with DMPs identified by the comparison between HD and septic patients. Gene ontology (GO) analysis of the shared (C) and not shared (D) DMPs from the previous representation. E Violin plot of the mean methylation status of the identified DMPs with b-values obtained from monocytes derived from healthy donor PBMCs exposed in vitro for 4 days to interferon-alpha (IFNα) (100ng/mL), interferon gamma (IFNγ) (100ng/mL), tumor necrosis factor-alpha (TNFα) (10ng/mL) and untreated (Control) (n=3), [26]. Statistical significance: * p < 0.05, ** p < 0.01, *** p < 0.0001, **** p < 0.00001. [file 13073_2022_1137_MOESM8_ESM.pdf]

Figure S4

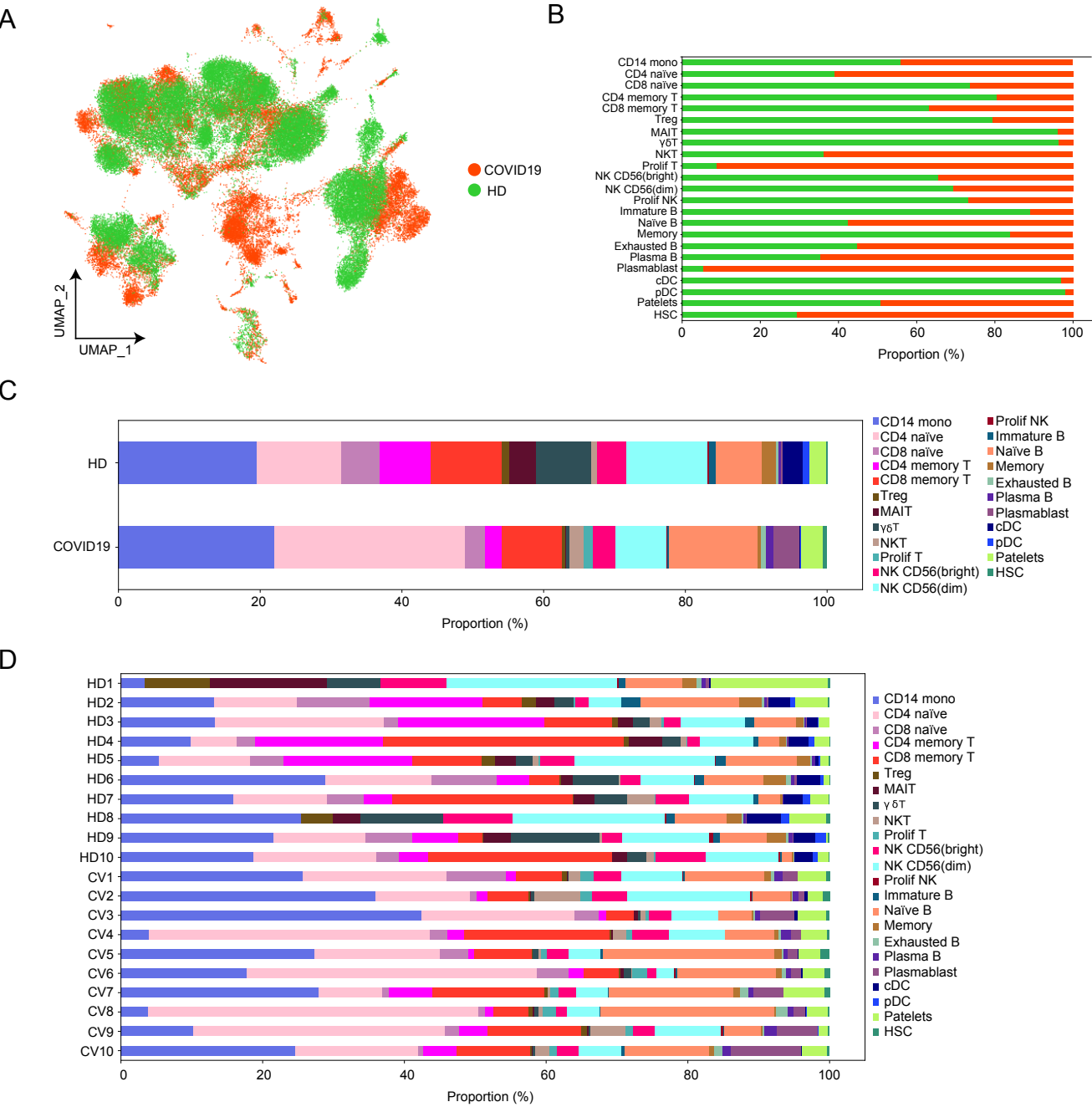

Supplement: Supplementary file 9 — Additional file 9: Figure S4. A UMAP visualization showing the two groups identified from Louvain clustering. B Barplot representation of the proportion of the two study groups in the different cell type found. C Barplot representation of proportion of each cell type in each study group. D Barplot representation of proportion of each cell type in each sample used for the scRNA-seq analysis. [file 13073_2022_1137_MOESM9_ESM.pdf]

Figure S5

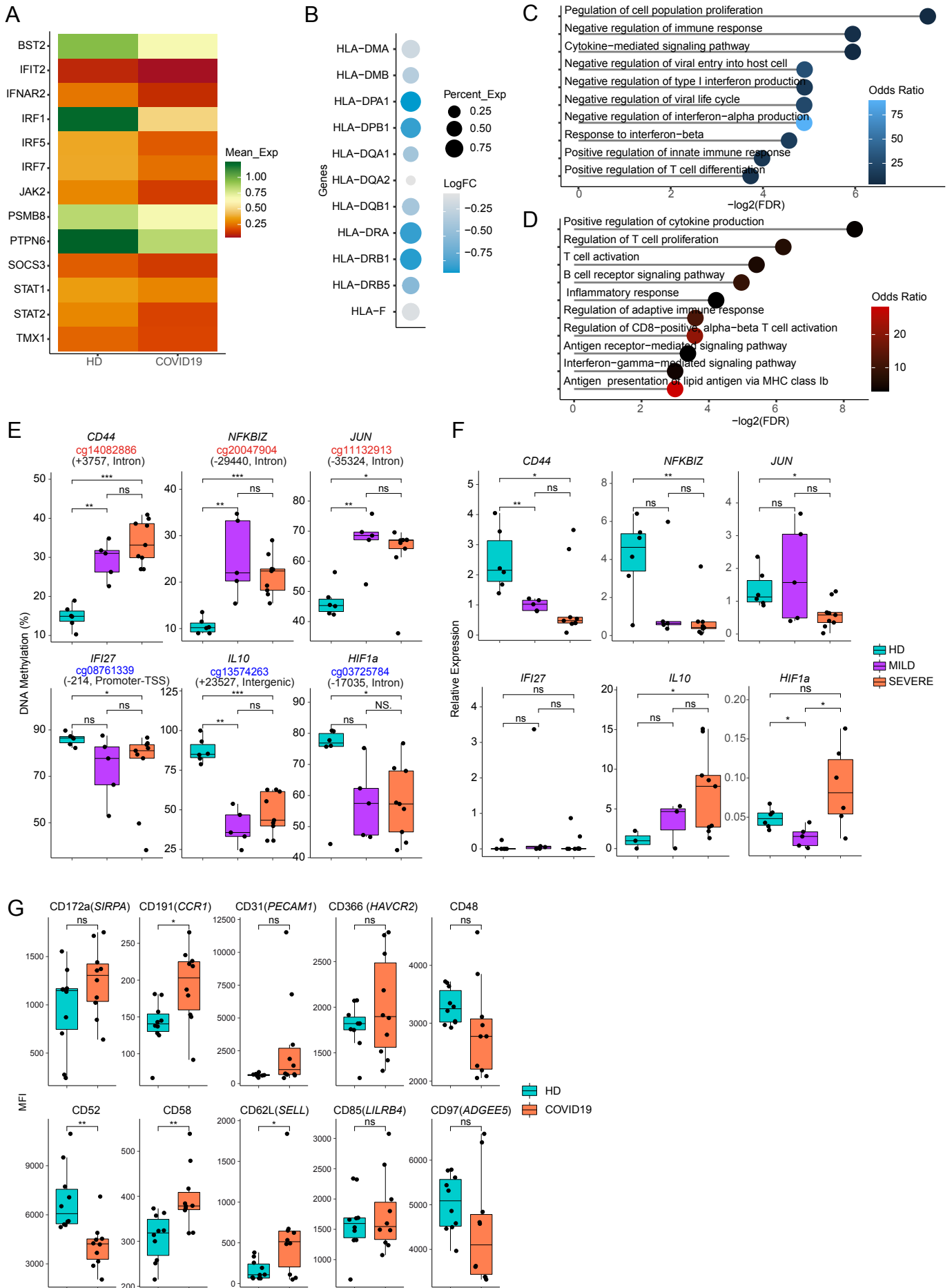

Supplement: Supplementary file 11 — Additional file 11: Figure S5. Analysis of DEG in monocytes derived from severe COVID-19 patients. A Heatmap representation of expression levels of IFN genes (ligands and receptor) in HD and COVID-19 CD14+ monocytes. Gene ontology (GO) analysis of the upregulated. B Dot plot of major histocompatibility complex (MHC) genes. LogFc and percentage of expression is representeate. C and downregulated (D) DEG that present a negative correlation with their close CpG. E Box plot of individual DNA methylation values of CpG from the hypermethylated and hypomethylated clusters with the name of the closest gene and the position in respect to the transcription start site. Calculated using pyrosequencing in the validation cohort that include HD and patients with mild and severe infection of COVID-19. F Box plot of relative expression of individual genes performed by real-time quantitative polymerase chain reaction (RT-qPCR) in the validation cohort that include HD and patients with mild and severe infection of COVID-19. G Box plot of mean florescence intensity (MFI) of cell surface markers in CD14+ cells in the cohort used for single cell analysis. Statistical significance: * p < 0.05, ** p < 0.01, *** p < 0.0001, **** p < 0.00001. [file 13073_2022_1137_MOESM11_ESM.pdf]
